# Phase 1, placebo-controlled, dose escalation trial of chicory root extract in patients with osteoarthritis of the hip or knee

**DOI:** 10.1186/1471-2474-11-156

**Published:** 2010-07-09

**Authors:** Nancy J Olsen, Valerie K Branch, Geetha Jonnala, Mira Seskar, Melisa Cooper

**Affiliations:** 1 Rheumatic Diseases Division, Department of Internal Medicine, University of Texas Southwestern Medical Center, 5323 Harry Hines Blvd., Dallas TX 75390 USA; 2 Phytomedics, Inc., 1085 Cranbury South River Road Suite #8, Jamesburg NJ 08831 USA; 3 Division of Rheumatology, 500 University Drive, Penn State Hershey Medical Center, Hershey PA 17033 USA

## Abstract

**Background:**

Extracts of chicory root have anti-inflammatory properties *in vitro *and in animal models of arthritis. The primary objective of this investigator-initiated, Phase 1, placebo-controlled, double blind, dose-escalating trial was to determine the safety and tolerability of a proprietary bioactive extract of chicory root in patients with osteoarthritis (OA). Secondary objectives were to assess effects on the signs and symptoms of this disorder.

**Methods:**

Individuals greater than 50 years of age with OA of the hip or knee were eligible for trial entry. A total of 40 patients were enrolled in 3 cohorts and were treated with escalating chicory doses of 600 mg/day, 1200 mg/day and 1800 mg/day for 1 month. The ratio of active treatment to placebo was 5:3 in cohorts 1 and 2 (8 patients) each and 16:8 in cohort 3 (24 patients). Safety evaluations included measurement of vital signs and routine lab tests at baseline and the end of the treatment period. Efficacy evaluations at baseline and final visits included self-assessment questionnaires and measurement of the 25-foot walking time.

**Results:**

In the highest dose cohort, 18 patients who completed treatment per protocol were analyzed for efficacy. In this group, 13 patients showed at least 20% improvement in the defined response domains of pain, stiffness and global assessment: 9 of 10 (90%) patients randomized to active treatment with chicory and 4 of 8 (50%) patients randomized to placebo (P = 0.06). In general, the treatment was well-tolerated. Only one patient who was treated with the highest dose of chicory had to discontinue treatment due to an adverse event.

**Conclusions:**

The results of this pilot study suggest that a proprietary bioactive extract of chicory root has a potential role in the management of OA and merits further investigation. Clinicaltrials.gov identifier: NCT 01010919.

## Background

Osteoarthritis (OA) is the most common form of arthritis and it is increasing in prevalence as the population ages [1]. Disability costs related to OA are significant and this condition is the most common indication for joint replacement of the hip or knee [2]. Treatment regimens for OA alleviate symptoms but do not modify the course of the disease. The most frequently used agents are nonsteroidal anti-inflammatory drugs (NSAIDs) and acetaminophen. Controlled studies have suggested that treatments that have anti-inflammatory properties provide greater symptom relief than analgesics alone [3]. However, the NSAIDs that are usually used have adverse effects [4] and in recent years the use of Cox-2-selective agents has been shown to be associated with an increased risk of cardiovascular morbidity and mortality [5]. These treatment issues have led to increased interest in alternative therapies that might offer relief of symptoms with fewer side effects. Nutritional supplements of many types have been utilized for arthritis, and those containing glucosamine and chondroitin sulfate have been shown to provide pain relief [6]. Other supplements have been tried, but evidence in favor of their use is generally limited to anecdotal experience or *in vitro *effects [7,8].

We were interested in evaluating effects of a proprietary bioactive extract of chicory root in patients with OA, based on *in vitro *data demonstrating its anti-inflammatory properties including inhibition of the production of COX-2 [9], iNOS, TNF-α and NFκB [9,10]. Pre-clinical toxicology studies have shown no adverse effects in rodents [10]. Chicory has a long history of use for other medical conditions such as digestive disorders, it has for many years been added to food products like coffee and it has a very good safety profile. If chicory could alleviate some of the signs and symptoms of OA while having fewer side effects than NSAIDs, we postulated that it would have clinical utility.

The present study was designed to evaluate safety and tolerability of treatment with a proprietary bioactive extract of chicory root in patients with OA of the hip or knee evaluated over a treatment period of 1 month. This design was based on clinical observations that NSAIDs generally show beneficial effects over a similar timeline. Previous studies in OA have shown relatively high placebo effects, and therefore the design of this study was double blind with a placebo control group. Patients were enrolled into 3 separate cohorts, with stepwise increases in the daily dose of the active agent.

## Methods

### Patients and protocol requirement

To be included in this study patients had to be 50 years of age or older and have an imaging-confirmed (radiograph or MRI) diagnosis of OA of the hip or knee. The use of an NSAID was not permitted in the 7 days prior to the enrollment visit, and intra-articular steroids could not have been administered within the previous 30 days. In addition, no enrolled patient had received intra-articular hyaluronate injections in the month prior to enrollment. One individual who had been treated with hyaluronate in the past had noted no benefit from the series of injections. Patients had to be able to ambulate sufficiently to complete a 25 foot walking time. Individuals with significant active medical conditions were excluded at the discretion of the principal investigator. The protocol and the case report forms were written entirely by the investigators (NO, VB and GJ). The protocol was approved by the University of Texas Southwestern Institutional Review Board and informed consent was obtained from all individuals prior to the baseline study visit activities. Clinicaltrials.gov identifier is NCT 01010919.

At the baseline visit, a medical history and physical exam were carried out. The patient completed the following self-assessment questionnaires:

McMaster Universities Osteoarthris Index (WOMAC)[11,12]

Brief Pain Inventory (BPI)[13]

Modified health assessment questionnaire (MHAQ)[14]

Visual analog scale (10 cm) for arthritis pain assessment (VAS)

Each patient also was timed for a 25 foot walk. The following laboratory tests were obtained and sent to the local clinical laboratory: complete blood count, AST, ALT and creatinine. Blinded medication provided by the study sponsor was dispensed. One batch of chicory, chemically verified for content by a third party laboratory, was used for all subjects. Randomization of the doses was pre-determined and built into the packaging, so that each numbered box of medication was dispensed in sequence to each enrolled patient. The dispensed capsules each contained either 200 mg of chicory extract or matching placebo.

Three study cohorts were enrolled. The first cohort took one 200 mg capsule three times daily (TID) for a total dose of 600 mg/day. The second cohort took 2 capsules TID for a total dose of 1200 mg/day and the third cohort took 3 capsules TID for a total daily dose of 1800 mg/day. Patients randomized to placebo took matching placebo capsules on the same schedule. To encourage enrollment, the ratio of active treatment to placebo was 5:3 in cohorts 1 and 2 and 16:8 in cohort 3. One patient assigned to cohort 3 (and randomized to placebo) took 1 capsule three times daily rather than 3 capsules three times daily and had 100% compliance with this regimen. Therefore, blinded data on this individual were analyzed with cohort 1. With this change in assignment, the final breakdown of treatment was as follows:

Cohort 1 - 9 Patients: 4 placebo, 5 chicory

Cohort 2 - 8 Patients: 3 placebo, 5 chicory

Cohort 3 - 23 Patients: 8 placebo, 15 chicory

Patients were given diaries to record dosing and use of the permitted rescue analgesics, acetaminophen alone or combined with either hydrocodone or codeine. The final visit was scheduled 4 weeks after the baseline and at this visit the same tests, instruments and assessments were carried out. Pill counts were completed and patients were discharged from the study.

### Efficacy assessment and analysis

Efficacy was determined in three domains. The first was pain, measured as change from baseline in one of the following: WOMAC question #1, WOMAC question #2, or the mean of items 3,5 and 6 on the BPI. The second domain was stiffness, measured as change from baseline in at least one of the following: WOMAC question #3, #4 or #5. The third domain was global functional assessment measured as change from baseline in walking time, mHAQ score or the mean of BPI in questions 9a-9g. Improvement in each domain was defined as a change of at least 20% compared to the pretreatment baseline, analogous to the ACR20 response criteria for rheumatoid arthritis. The efficacy analyses were done with the blinded data; treatments were not unblinded until after each individual subject was assigned improved or unimproved status (by NO) and this status was confirmed (by MC). Only after this determination was complete were the treatment assignments unblinded.

### Safety analysis

Changes in laboratory parameters and vital signs were analyzed by comparing baseline and final visits for each individual subject.

### Statistics

Analysis for efficacy was done using the per protocol population. Safety analyses included all patients who received any dose of blinded study medication. For continuous variables, data were expressed as the mean and standard error of the mean (SEM). Groups of continuous variables were compared using a t-test for 2 groups or a 1-way ANOVA for 3 or more groups. Baseline and final safety variables were compared using a paired t-test. Discontinuous variables were compared using a chi-square test. For all statistical analysis, P values < 0.05 were considered significant.

## Results

### Patient Characteristics

The 40 enrolled patients had an average age of 63 years and included predominantly females (N = 30) and non-Hispanic Caucasians (N = 33). The mean BMI for enrolled subjects was 31, consistent with moderate obesity. There were no significant differences between the three study groups in these demographic variables (Table 1). Although many patients had arthritis in both the hip and knee, each subject was asked to identify the one joint area that currently caused the most problems, and for the majority of patients (N = 33) this was the knee. Indicators of disease severity and pain were somewhat worse in cohort 1, and these differences were statistically significant for the walking time (P = 0.009) and the average score on WOMAC Item #5 (p = 0.047). Baseline laboratory tests were all within normal ranges and were not significantly different between groups, with the exception of hemoglobin levels, which were lower in cohort 1 (P = 0.01). All of the participants in cohort 1 were female, which may have contributed to this result.

**Table 1:** Demographic and disease features at baseline in 3 cohorts

VARIABLES	COHORTS			P VALUES*
	**1**	**2**	**3**	

Age (years)	59 ± 2	64 ± 4	65 ± 2	0.28

Gender F/M	9/0 (100%)	5/3 (62.5%)	16/7 (70%)	0.133

Non-Hispanic Caucasian	67%	75%	91%	0.21

BMI	35.3 ± 4.6	30.4 ± 1.6	29.6 ± 1.2	0.20

Knee/Hip	8/1	5/3	20/3	0.60

Walking Time (sec)	12.0 ± 2.6	7.3 ± 0.7	7.7 ± 0.4	0.009

WOMAC Item #1	3.2 ± 0.3	2.4 ± 0.3	3.9 ± 0.4	0.075

WOMAC Item #5	3.2 ± 0.3	2.4 ± 0.3	2.4 ± 0.2	0.047

BPI **	5.6 ± 0.9	2.6 ± 0.7	3.9 ± 0.4	0.19

Global VAS (mm)	54.5 ± 10.9	31.0 ± 6.7	45.8 ± 4.4	0.15

Modified HAQ (0-3 scale)	1.4 ± 0.2	0.7 ± 0.1	0.8 ± 0.1	0.17

Hemoglobin (g/dL)^†^	13.0 ± 0.5	14.2 ± 0.4	14.4 ± 0.2	0.016

Creatinine (mg/dL)^†^	0.77 ± 0.06	0.80 ± 0.09	0.82 ± 0.03	0.74

AST (U/L)^†^	19 ± 1	23 ± 2	24 ± 2	0.32

*P values for Age and BMI calculated with one-way ANOVA or Fisher's Exact Test.

**BPI Score is mean for items 3-6 and 9(A-G).

^† ^Normal ranges: Hemoglobin 13.2-16.2 g/dL; creatinine 0.7-1.2 mg/dL; AST 13-40 U/L.

Rates of study completion showed an increasing trend across cohorts: 5/9 (56%) in cohort 1, 6/8 (75%) in cohort 2 and 20/23 (87%) in cohort 3. In cohort 1, two patients withdrew early due to worsening arthritis pain and two were lost to followup. In cohort 2, two patients withdrew due to increased pain, and both were on chicory. No cohort 3 patients withdrew for worsening arthritis pain, but two were lost to followup and one discontinued treatment due to an adverse event (described below).

Some patients took permitted rescue analgesics during the study month. The most commonly used analgesic was acetaminophen, taken by one patient in cohort 1 who was assigned to chicory, one patient in cohort 2 who was also assigned to chicory and five patients in cohort 3, three on placebo and two on chicory. Narcotic analgesics, either hydrocodone or codeine in combination with acetaminophen, were taken for rescue therapy by two patients in cohort 1, both assigned to placebo, two in cohort 2, one each on chicory and placebo. No cohort 3 patient took narcotic analgesics for rescue. Three patients in cohort 1 took prohibited NSAIDs prior to the final visit due to increased pain, and these individuals (2 placebo, 1 chicory) were excluded from the efficacy analysis.

Compliance with treatment, defined as taking more than 80% of prescribed doses as evaluated at visit 2 (excluding patients who did not complete V2), was 83%, 86% and 86% in cohorts 1,2 and 3, respectively.

### Efficacy

**Figure 1:**
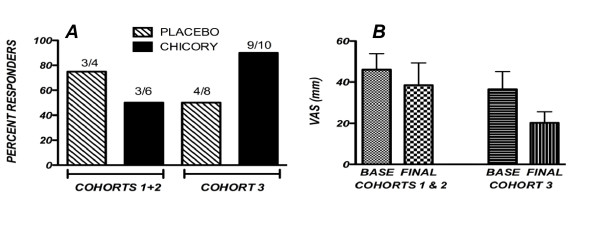
**(A) Percent responders in placebo and chicory groups and (B) Visual analog pain scores in at baseline and final visits in placebo and chicory treatment groups in Cohort 3**. (A) Individuals in cohorts 1 and 2 were combined for this analysis. The ratios over each bar indicate numbers of responders over total numbers of analyzed individuals in each study group. For Cohort 3, the difference between placebo and chicory responses had a corresponding P value of 0.06.

Efficacy data were analyzed for patients who had completed both visits without having taken any prohibited medications prior to either of the two assessments and who had at least an 80% compliance with the treatment regimen as determined by pill counts. Using these guidelines, numbers of analyzable patients were: 4 in cohort 1, 6 in cohort 2 and 18 in cohort 3. Improvement was defined as a positive change of at least 20% in at least 2 of the 3 domains (pain, stiffness and global). Using this definition, in cohort 1, 2 of 4 patients, 1 each on active treatment and placebo, were improved. In cohort 2, 4 of 6 were improved, 2 each on active treatment and placebo. In cohort 3, 13 patients were improved, 4 on placebo and 9 on chicory (Figure 1A). In the highest dose group, cohort 3, the difference between those improved on chicory (9/10; 90%) compared to those who improved on placebo (4/8; 50%) was close to statistical significance (P = 0.06). In this cohort, 6 of the 9 chicory responders showed improvement in both the pain and stiffness domains; two had response in the pain and global domains and one showed response in the stiffness and global domains. There was a trend for the visual analog measure of pain to be decreased in patients on active treatment in cohort 3, but this was not a statistically significant change (Figure 1B).

### Safety

Safety measures in patients treated with either chicory or placebo showed no clinically significant changes between initial and final determinations of vital signs (HR, BP) or laboratory measures (CBC, creatinine, AST/ALT) in any of the cohorts (Tables 2 and 3). A small and statistically significant change from baseline diastolic blood pressure (DBP) was observed in the cohort 3 chicory patients; DBP was elevated at baseline and improved to 90 mm Hg at the final visit (P = 0.044). Overall no safety concerns were raised by these analyses.

**Table 2:** Safety measures at visit 1 and visit 2 in patients receiving chicory

Variable Measured	Cohort 1			Cohort 2			Cohort 3		
	(N = 4)*			(N = 4)			(N = 13)		
	**Visit 1**	**Visit 2**	P**	**Visit 1**	**Visit 2**	**P**	**Visit 1**	**Visit 2**	**P**

BP Syst (mm Hg)	136 ± 9.3	128 ± 9.9	0.40	144 ± 7.8	141 ± 3.5	0.80	138 ± 5.7	142 ± 5.4	0.39

BP Diast (mm Hg)	83 ± 6.6	82 ± 5.5	0.86	78 ± 10.1	88 ± 3.7	0.46	96 ± 2.3	88 ± 3.4	0.04

HR (BPM)	69 ± 4.2	77 ± 4.6	0.01	74 ± 9.2	76 ± 9.4	0.74	72 ± 3.7	71 ± 4.3	0.65

WBC^† ^(cell/mm^3^)	6.1 ± 0.6	6.1 ± 0.3	0.48	6.6 ± 1.2	5.9 ± 0.9	0.20	6.9 ± 0.5	6.6 ± 0.2	0.34

Hgb (g/dL)	12.8 ± 0.8	12.6 ± 0.7	0.23	14.4 ± 0.9	14.5 ± 0.8	0.25	14.6 ± 0.2	14.1 ± 0.3	0.02

Creatinine (mg/dL)	0.78 ± 0.08	0.78 ± 0.07	1	0.68 ± 0.11	0.70 ± 0.11	0.39	0.83 ± 0.04	0.84 ± 0.04	0.67

AST (U/L)	17.3 ± 1.8	17.8 ± 0.5	0.81	24.0 ± 3.5	25.0 ± 5.0	0.57	21.9 ± 2.4	22.3 ± 2.4	0.86

* Patients included in this analysis were those receiving chicory who had lab values available at both baseline and final visits. Values represent mean and S.E.M.

** P values calculated using paired t-test, V1 vs V2.

^†^Normal range: WBC 4.1-11.1 × 10/μL.

**Table 3:** Safety measures at visit 1 and visit 2 in patients receiving placebo

Variable Measured	Cohorts 1&2 (N = 4)			Cohort 3 (N = 13)		
	**Visit 1**	**Visit 2**	P**	**Visit 1**	**Visit 2**	**P**

BP Syst (mm Hg)	124 ± 7.3	137 ± 5.9	0.14	129 ± 4.0	130 ± 5.7	0.78

BP Diast (mm Hg)	92 ± 8.6	90 ± 6.2	0.94	85 ± 2.4	85 ± 3.6	1.0

HR (BPM)	74 ± 6.0	66 ± 5.2	0.010	71 ± 4.1	74 ± 4.0	0.36

WBC (cell/mm^3^)	6.2*	5.9	--	7.1 ± 0.68	6.2 ± 0.67	0.002

Hemoglobin (g/dL)	14.1*	13.8	--	14.3 ± 0.3	13.8 ± 0.3	0.02

Creatinine (mg/dL)	0.8*	0.75	--	0.84 ± 0.06	0.86 ± 0.07	0.35

AST (U/L)	20.5*	21.5	---	23.9 ± 1.0	23.3 ± 5.1	0.56

* Only 2 placebo patients in cohorts 1,2 had paired blood samples, so SEM and P values could not be calculated.

Two patients reported adverse events. One patient in cohort 2, randomized to placebo, had intermittent nausea which did not interfere with completing the study or compliance and which did not require any therapeutic intervention. One patient in cohort 3, randomized to chicory, reported headache and diarrhea that started 5 days into the treatment course. This patient discontinued treatment after 10 days due to these problems and had complete resolution of all symptoms by the time of the final visit. The group assignment was not unblinded early in this individual.

## Discussion

OA remains a disorder without available disease-modifying treatments. Pain and limited function are the predominant manifestations of OA and these are currently managed with analgesic and anti-inflammatory medications and physical therapy or surgery, respectively. Due to the generally older age of OA patients and the high prevalence of comorbid conditions in this population, medical treatments are often limited by unacceptable side effects. Classic NSAIDs, many of which are now available over-the-counter, can cause gastrointestinal bleeding, fluid retention and blood pressure problems, and this limits therapeutic dosing in many patients. The COX-2 selective inhibitor drugs have lower rates of gastrointestinal complications, but carry increased risks of cardiovascular adverse events. These concerns have led to updated guidelines from the American Geriatrics Society suggesting that anti-inflammatory drugs should not be used on a chronic basis in treating patients 75 years of age or older [15]. Analgesics without anti-inflammatory properties, notably acetaminophen, are often recommended as a safer alternative. However, many OA patients in clinical practice find acetaminophen to be generally less effective than NSAIDs, a finding that also has been confirmed in controlled clinical trials [3]. Acetaminophen in addition carries risks of liver toxicity [16]. A consequence of this situation with available medications is that many patients with OA do not have adequate control of the pain associated with their disease. Because reduction in ambulatory activities that might result from hip and knee OA can have other health impacts, poor pain control is likely to result in a significant overall functional decline.

Alternative approaches using various nutraceuticals as potential arthritis therapeutics, including plant-derived agents with anti-inflammatory properties, have been advocated [17,18]. This concept is based in part on the concept that the health of joint cartilage is dependent upon the availability of nutritional factors including essential fatty acids, antioxidants, vitamins and minerals [7]. Chicory root extract is of interest because it has been shown to suppress the production of key cytokines and enzymes involved in inflammation. Preliminary studies with chicory carried out in the collagen-induced murine arthritis model showed that this agent reduced joint swelling with a magnitude similar to the NSAID indomethacin (unpublished data, Phytomedics).

Limitations of the current trial include the short duration of treatment which may have been insufficient to demonstrate maximal efficacy as well as to detect adverse events resulting from chronic treatment. A mixed population of OA patients was enrolled, including different sites and use of a per-protocol population rather than an intention-to-treat (ITT) design, could have introduced significant biases and gives a less conservative estimate of treatment effect. The WOMAC measures of outcome did not follow previously-validated approaches for this instrument. Future trials will be designed to include a longer treatment period, more restricted entry criteria for OA features, validated OA outcome measures (e.g. OARSI), and ITT analyses to confirm the potential utility of this treatment.

Data generated in the present small pilot trial suggest that a proprietary bioactive extract of chicory root might be efficacious in patients with OA of the hip or knee. Although the approach used to assess efficacy has not been validated, this trial is comparable in some respects to the previously-reported large study of glucosamine and chondroitin sulfate in which the primary outcome was a 20% reduction in knee pain [6]. Demographic characteristics were similar in the two studies, and the range of placebo responses in our patients (50-75%) was comparable to the 60% rate reported in this previous trial.

## Conclusions

These preliminary results are encouraging and will require verification in a larger study with longer treatment duration to confirm safety and efficacy of this proprietary extract of chicory root for the treatment of OA as monotherapy or in combination with low doses of NSAIDs.

## Competing interests

NJO received a research grant from Phytomedics to design and conduct this study; MS and MC are employees of Phytomedics.

## Authors' contributions

NJO wrote the protocol, VKB coordinated recruitment and enrollment of study subjects, GJ designed the case report forms, MS was responsible for the randomized medication kits and MC designed the outcome analysis. NJO and MC coordinated manuscript writing and editing with the cooperation of all coauthors. All authors read and approved the final manuscript.

## Pre-publication history

The pre-publication history for this paper can be accessed here:

http://www.biomedcentral.com/1471-2474/11/156/prepub
